# The Impact of the COVID-19 Pandemic on the Mental Health of Healthcare Workers in Italy: Analyzing the Role of Individual and Workplace-Level Factors in the Reopening Phase After Lockdown

**DOI:** 10.3389/fpsyt.2022.867080

**Published:** 2022-06-02

**Authors:** Maria Francesca Moro, Gemma Calamandrei, Ranieri Poli, Valentina Di Mattei, Alessandra Perra, Peter Konstantin Kurotschka, Alexandra Restrepo, Ferdinando Romano, Giuseppe La Torre, Emanuele Preti, Franco Mascayano, Angelo Picardi, Flavia Chiarotti, Venerando Rapisarda, Antonio Urban, Ruben Alvarado, Ezra Susser, Mauro Giovanni Carta

**Affiliations:** ^1^Department of Epidemiology, Mailman School of Public Health, Columbia University, New York, NY, United States; ^2^University of Cagliari, Cagliari, Italy; ^3^Istituto Superiore di Sanità,Rome, Italy; ^4^Azienda Ospedaliera Universitaria Integrata di Verona, Verona, Italy; ^5^Università Vita Salute San Raffaele Milano, Milan, Italy; ^6^Department of General Practice, University Hospital Wuerzburg, Wuerzburg, Germany; ^7^Universidad de Antioquia, Medellín, Colombia; ^8^Dipartimento di Sanità Pubblica e Malattie Infettive, Università La Sapienza, Rome, Italy; ^9^Università degli Studi Milano-Bicocca, Milan, Italy; ^10^New York State Psychiatric Institute, NewYork, NY, United States; ^11^University of Catania, Catania, Italy; ^12^Azienda Ospedaliero Universitaria di Cagliari, Cagliari, Italy; ^13^Department of Public Health, School of Medicine, Faculty of Medicine, Universidad de Valparaiso, Valparaíso, Chile

**Keywords:** healthcare workers, COVID-19, mental health, stigma, violence, depression, psychological distress, Italy

## Abstract

**Introduction:**

Italy is one of the high-income countries hit hardest by Covid-19. During the first months of the pandemic, Italian healthcare workers were praised by media and the public for their efforts to face the emergency, although with limited knowledge and resources. However, healthcare workers soon had to face new challenges at a time when the national health system was working hard to recover. This study focuses on this difficult period to assess the impact of the COVID-19 pandemic on the mental health of Italian healthcare workers.

**Materials and Methods:**

Healthcare workers from all Italian regions [*n* = 5,502] completed an online questionnaire during the reopening phase after the first wave lockdown. We assessed a set of individual-level factors (e.g., stigma and violence against HCWs) and a set of workplace-level factors (e.g., trust in the workplace capacity to handle COVID-19) that were especially relevant in this context. The primary outcomes assessed were score ≥15 on the Patient Health Questionnaire-9 and score ≥4 on the General Health Questionnaire-12, indicators of clinically significant depressive symptoms and psychological distress, respectively. Logistic regression analyses were performed on depressive symptoms and psychological distress for each individual- and workplace-level factor adjusting for gender, age, and profession.

**Results:**

Clinically significant depressive symptoms were observed in 7.5% and psychological distress in 37.9% of HCWs. 30.5% of healthcare workers reported having felt stigmatized or discriminated, while 5.7% reported having experienced violence. Feeling stigmatized or discriminated and experiencing violence due to being a healthcare worker were strongly associated with clinically significant depressive symptoms [OR 2.98, 95%CI 2.36–3.77 and OR 4.72 95%CI 3.41–6.54] and psychological distress [OR 2.30, 95%CI 2.01–2.64 and OR 2.85 95%CI 2.16–3.75]. Numerous workplace-level factors, e.g., trust in the workplace capacity to handle COVID-19 [OR 2.43, 95%CI 1.92–3.07] and close contact with a co-worker who died of COVID-19 [OR 2.05, 95%CI 1.56–2.70] were also associated with clinically significant depressive symptoms. Similar results were found for psychological distress.

**Conclusions:**

Our study emphasizes the need to address discrimination and violence against healthcare professionals and improve healthcare work environments to strengthen the national health system's capacity to manage future emergencies.

## Introduction

Italy is one of the high-income countries hit hardest by the Covid-19 pandemic, showing a mortality rate for SARS-CoV-2 among the highest in the world during the first wave in 2020 (1, 2). When the first cases of COVID-19 were confirmed in the country on 31 January 2020, Italy was preparing to face the pandemic with confidence (3). Despite the growing alarm for the decreases in the expenses allocated to the national healthcare system and the protests by citizens and service users' organizations against these cuts over the past years (4–6), official voices stated with pride that the Italian healthcare system was “one of the best in the world based on authoritative rankings,” and it would have been well prepared to give a right answer to the threat (5, 7).

The pandemic has revealed the excessive optimism of these statements and has produced a shocking awareness in healthcare workers (HCWs) of the fragility and vulnerability of the Italian healthcare system (8). During the first months of the pandemic, a substantial number of health professionals found themselves without basic personal protective equipment, with scarce opportunities to get tested for the virus, and with the additional challenges of working in a system with considerable organizational deficiencies that soon went into crisis (9–13). In 2020, according to the Italian National Institute for Insurance against Accidents at Work, around 70% of over 100,000 work accidents claims due to COVID-19 were from HCWs, and 60% of the fatal cases concerned nurses (14). This situation heavily affected the mental wellbeing of Italian HCWs, as shown in previous studies. Using data collected during the first months of the pandemic, Lasalvia et al. (11) reported that 53.8% [95% CI (51.0–56.6%)] of the HCWs in their study showed symptoms of post-traumatic distress, 50.1% (95% CI [47.9–52.3%]) symptoms of clinically relevant anxiety and 26.6% [95% (CI 24.7–28.5%)] of at least moderate depression. Similarly, Rossi et al. (15) conducted a study among HCWs during the first wave of the pandemic and found that 49.4% of the respondents showed symptoms of post-traumatic stress disorder, 24.7% symptoms of depression, and 19.8% symptoms of anxiety.

On 18 May 2020, the national lockdown, which heavily restricted the movement of the population except for necessity, work, and health circumstances for 69 days, was lifted. Although this was seen with joy by the general population, it raised concerns among HCWs, who were worried that reopening prematurely after the first wave could be a recipe for disaster. There were also concerns that the National Health System would not be able to cope with a new wave at a time when staff was working hard to recover. Moreover, while during the first months of the pandemic HCWs were praised by the media and the public and even applauded from balconies as “heroes,” this quickly changed after the reopening. Anger began to ferment in the community against the inefficiencies of the national healthcare system and HCWs, although victims themselves of these inefficiencies, began to be mistakenly seen as the culprits of the failures in addressing COVID-19. In a few weeks, they went from heroes to negligent professionals in the eyes of many persons, while a number of lawyers and prosecutors started to put their decisions and treatment choices under judiciary scrutiny (16). Although studies from other countries have shown the negative impact of stigma and violence related to COVID-19 on HCWs' mental wellbeing (17, 18), these factors have been neglected in previous research conducted in Italy. In the present paper, we used a large national sample of Italian HCWs to examine whether their mental wellbeing during the challenging reopening phase after lockdown was related to 1) individual-level factors such as stigma and violence against HCWs, and 2) workplace-level factors related to COVID-19.

## Materials and Methods

### Study Design

This cross-sectional study is part of a prospective international project entitled “The COVID-19 HEalth caRe wOrkErS (HEROES) study” (NCT04352634) (19), which includes participants from 25 countries across 4 continents and was jointly launched by Columbia University Mailman School of Public Health and Universidad de Chile School of Public Health (Faculty of Medicine), in collaboration with the Pan American Health Organization (PAHO) and with support from the World Health Organization (WHO). In Italy, the study was supported by the National Institute of Health.

Data were collected in all Italian regions during the reopening phase after the first wave lockdown, from May 2020 to July 2020 (follow-up assessments will be carried out at 18 and 24 months). Participants were clinical and non-clinical HCWs employed in in-patient or out-patient health facilities throughout the country. They were recruited through an invitation sent via email. A list of emails was previously obtained from: (1) HCWs national and/or local orders and organizations (the involvement of orders and organizations allowed to recruit health professionals working at the community level, such as general practitioners and HCWs employed in retirement homes and other facilities outside of the hospital system but heavily affected by COVID-19), (2) HCWs national and/or local unions (the involvement of unions allowed to recruit HCWs usually not organized in orders and organizations, such as cleaning staff, maintenance staff, and others), (3) selected health centers and hospitals. Particular attention was given to involve orders, organizations, unions, health centers, and hospitals and recruit participants from Italian areas differently affected by the COVID-19 pandemic in the North, Center, and South of the country. Further details on the sampling procedures can be found in the study protocol (19).

### Measures

An on-line self-administered questionnaire including standardized measures and some items created *ad hoc* was used for data collection. The questionnaire included the following sociodemographic variables: gender, age, region, and members of household under 18 years, over 65 years, and with disabilities.

To assess mental wellbeing, we evaluated depressive symptoms and psychological distress. Depressive symptoms were measured by the Italian version of the 9-items Patient Health Questionnaire (PHQ-9) (20, 21). Total PHQ-9 scores range from 0 to 27. According to these scores, depression severity is categorized as “none or minimum” (0-4), “mild” (5-9), “moderate” (10-14), “moderately severe” (15-19), and “severe” (20-27). In this study, PHQ-9 scores ≥ 15 were categorized as indicative of clinically significant depressive symptoms (CSD), which suggest a high probability of major depression for which treatment is recommended (21). Psychological distress was measured by the Italian version of the General Health Questionnaire 12-Items (GHQ-12) (22, 23). Total GHQ-12 scores were calculated using the bimodal scoring method [0-0-1-1]. According to this method, GHQ-12 scores ≥ 4 were categorized as indicative of clinically significant psychological distress (GHQ+) (24). Cronbach's alphas were 0.87 for the PHQ-9 and 0.88 for the GHQ-12.

Individual-level covariates were measured as follows: Feeling stigmatized or discriminated against for the role as HCWs due to the pandemic (Agree, Disagree), Having experienced violence due to being a HCWs during the pandemic (Agree, Disagree), Fear of getting COVID-19 (Yes, No), Fear of infecting the loved ones (Yes, No), Having a loved one infected with COVID-19 (Yes, No), Having loved ones who provided support when needed (Yes, No). Workplace-level covariates were measured as follows: Days in isolation due to COVID-19 (0, 1-19, ≥ 20), hours worked per day on average (≥ 10, ≤ 9), Assignment to a new team or functions (Yes, No), Lack of masks at work (Yes, No), Close contact with patients who were suspected or confirmed cases of COVID-19 (Yes, No, I don't know), Close contact at work with someone who passed away due to COVID-19 (Yes, No, I don't know), Having cared for patients with COVID-19 that passed away (Yes, No, I don't know), Trust in the workplace capacity to manage COVID-19 (low, high), Having a reliable network of supportive colleagues at work (Agree, Disagree). Further details on variables included in the study can be found in Table 1 and the study protocol (19).

**Table 1:** Variables from the HEROES questionnaire used in this study.

**Construct**	**Variables**	**Answers**
Sociodemographic	- Age - Gender (Woman/Man/Other gender) - Region - Members of household under 18 years, over 64 years, and with disabilities (Yes vs. No) - Profession	Ad**-**hoc questions
Experiences, fears, and concerns about COVID-19 (Individual-level factors)	- Days of isolation for COVID-19 (continuos) - Fear of transmitting COVID-19 to loved ones (Likert scale recategorized as Yes/No) - Fear of being infected (Likert scale recategorized as Yes/No) - Having a loved one infected by COVID-19 (yes/no) - Having a loved one passing away due to COVID-19 (yes/no) - Feeling stigmatized as a health worker (Likert scale recategorized as Agree/Disagree) - Experiencing violence due to be a health worker (Likert scale recategorized as Agree/Disagree) - Support from loved ones (Likert scale recategorized as Agree/Disagree)	Ad**-**hoc questions
Work environment (Workplace-level factors)	- Change of functions since the start of the pandemic (yes/no) - Amount worked in the past week (hours) - Lack of masks (Likert scale recategorized as yes/no) - Contact with patients with COVID- 19 (yes/ I do not know / no) - Experience with the death of colleagues or patients with COVID-19 (yes / I do not know / No) - Trust in the workplace (Likert scale recategorized as low/high) - Support from colleagues (Likert scale recategorized as Agree/Disagree)	Ad**-**hoc questions
Psychological distress	Distress symptoms	General Health Questionnaire (GHQ**-**12)
Depression	Depressive symptoms	Patient Health Questionnaire (PHQ-9)

### Analyses

First, respondents who did provide informed consent but did not continue the survey or did not work in a facility providing healthcare were excluded. Then, respondents who did not complete the survey questions on depressive symptoms and psychological distress were removed. Sensitivity analyses were performed to evaluate potential differences between completers and non-completers. Tabular analyses were used to examine clinically significant depressive symptoms and psychological distress by assessing frequencies for the whole sample and stratified by gender, age, and profession. To examine the association between the individual and workplace-level variables of interest shown in Table 1 (selected a priori from the literature or based on clinical and empirical grounds) on HCWs mental wellbeing, we conducted logistic regression models on depressive symptoms and psychological distress for each variable while controlling for gender, age, and profession. Results were presented as odds ratios (ORs) with 95% confidence intervals. All analyses were performed using SAS (version 9.4 for Windows).

## Results

### Characteristics of the Respondents

Overall, 5,502 healthcare workers were recruited at baseline. Eighty-nine respondents were excluded because they did provide informed consent but did not continue the survey or did not work in a facility providing healthcare (information on non-responders non-available). Then 1,093 respondents who did not complete the survey questions on depressive symptoms and psychological distress were removed (*n* = 1,093). In total, 4,320 healthcare workers (78.5%) completed the survey questions on depressive symptoms and psychological distress. There were no relevant differences between completers and non-completers regarding gender (completers: woman 70.8%, man 29.1, non-completers: woman 67.0, man 26.4) and median age (completers: 45, non-completers: 44). Questions on depressive symptoms and psychological distress were in the last section of the questionnaire, suggesting that missingness was mainly at random and due to the length of the questionnaire.

Tables 2–4 show respondents' characteristics, with clinically significant depressive symptoms (CSD) stratified by gender (Table 2), age (Table 3), and profession (Table 4). Overall, 70.79% of participants identified as women, 29.12% as men, and 0.09% as other gender. The median age was 45. Most respondents were physicians (35.53%), nurses (12.55%), occupational therapists, educators, and rehabilitation technicians (8.96%), physical therapists (8.24%), speech therapists (4.72%), laboratory technicians (4.19%), and other staff, including pharmacists, healthcare assistants, dental hygienists, public health workers, and surveillance personnel (11.32%). Clinically significant depressive symptoms were reported by 326 participants, 7.55% of the overall sample. Women were more likely than men to report CSD [*OR* = 1.71 95% CI (1.29-2.27)]. When comparing the different professions, only nurses reported higher CSD than physicians [OR = 1.60: 95%CI (1.15–2.22)]. Regarding psychological distress, 1,637 respondents (37.89% of the overall sample) screened positive at the GHQ-12, with women reporting higher psychological distress than men [*OR* = 1.56: 95% CI (1.36–1.89)]. Supplementary Materials 1–3 show respondents' characteristics, with clinically significant psychological distress stratified by gender (Supplementary Material 1), age (Supplementary Material 2), and profession (Supplementary Material 3). In addition, 30.51% of the participants reported having felt stigmatized or discriminated for their role as HCWs and 5.72% reported having experienced violence due to being a healthcare worker during the pandemic.

**Table 2:** Characteristics of the respondents: CSD stratified by gender.

	**CSD (%)**	**No CSD (%)**	**Total (%)**	**OR (95% CI)**
Man	65	1,193	1,258 (29.12)	—
Woman	261	2,797	3,058 (70.79)	**1.71 (1.29-2.27)**
Other gender	0	4	4 (0.09)	**
Total	326 (7.55)	3,994 (92.45)	4,320 (100)	

**Not calculable.

**Table 3:** Characteristics of the respondents: CSD stratified by age.

	**CSD (%)**	**No CSD (%)**	**Total (%)**	**OR (95% CI)**
≤ 30	61	740	801 (18.54)	—
31-43	94	1,177	1,271 (29.42)	0.97 (0.69–1.35)
44-56	116	1,243	1,359 (31.46)	1.13 (0.82–1.56)
57-69	53	810	863 (19.98)	0.79 (0.54–1.16)
≥70	2	24	26 (0.60)	1.01 (0.23–4.38)
Total	326 (7.55)	3,994 (92.45)	4,320 (100)	

**Table 4:** Characteristics of the respondents: CSD stratified by profession.

	**CSD (%)**	**No CSD (%)**	**Total**	**OR (95% CI)**
Physician	113	1,422	1,535 (35.53)	—
Social worker	0	5	5 (0.12)	**
Occupational therapist/educator/ rehabilitation technician	24	363	387 (8.96)	0.83 (0.53–1.31)
Physical therapist	30	326	356 (8.24)	1.16 (0.76–1.76)
Nurse	61	481	542 (12.55)	**1.60 (1.15–2.22)**
Speech therapist	13	191	204 (4.72)	0.86 (0.47–1.55)
Dietician	1	26	27 (0.63)	0.48 (0.06–3.60)
Socio-sanitary operator	7	86	93 (2.15)	1.02 (0.46–2.27)
First responder (e.g., EMT)	1	12	13 (0.30)	1.05 (0.14–2.27)
Dentist	4	60	64 (1.48)	0.84 (0.12–6.90)
Midwife	1	14	15 (0.35)	0.90 (0.12–6.90)
Psychologist	4	76	80 (1.85)	0.66 (0.24–1.84)
Administrator/secretary/admissions/patient information	7	108	115 (2.66)	0.82 (0.37–1.43)
Clinical and non-clinical manager (director)	3	38	41 (0.95)	0.99 (0.30–3.27)
Laboratory technician	10	171	181 (4.19)	0.74 (0.38–1.43)
Maintenance, food, and security staff	1	17	18 (0.42)	0.74 (0.10–561)
Cleaning staff	1	25	26 (0.60)	0.50 (0.07–3.75)
Radiology technician	10	116	126 (2.92)	1.08 (0.55–2.13)
Other staff	35	454	489 (11.32)	0.97 (0.65–1.44)
Total	326 (7.55)	3,991 (92.45)	4,317 (100)	

*Missing data (n = 3)*.

**Not calculable.

### Association Between Healthcare Workers Individual- and Workplace-Level Factors and Clinically Significant Depressive Symptoms

Table 5 summarizes the results from regression analyses on depressive symptoms for individual-level factors. Feeling stigmatized or discriminated and experiencing violence due to being a HCW during the pandemic increased the likelihood of reporting CSD [*OR* = 2.98: 95% CI (2.36–3.77)] and [*OR* = 4.72: 95% CI (3.41–6.54), respectively]. Participants were more likely to report clinically significant depressive symptoms if they lived with persons older than 64 years [*OR* = 1.50: 95% CI (1.08–2.09)] or with disabilities [*OR* = 3.08: 95% CI (2.09–4.54)], or if they had a loved one infected with COVID-19 (*OR* = 1.78: 95% [CI 1.13–2.82]). On the contrary, having loved ones able to provide support when needed was a protective factor [*OR* = 0.26: 95% CI (0.20–0.35)].

**Table 5:** Association between health workers individual-level factors and CSD (PHQ-9 ≥ 15) or GHQ+ (GHQ-12 ≥ 4).

	**OR (95% CI)**	
	**CSD**	**GHQ+**
Living with persons younger than 18 years: Yes vs. No	0.89 (0.68–1.17)	0.90 (0.78–1.05)
Living with persons older than 64 years: Yes vs. No	**1.50 (1.08–2.09)**	**1.22 (1.01–1.49)**
Living with persons with disabilities: Yes vs. No	**3.08 (2.09–4.54)**	**1.94 (1.46–2.56)**
Fear of getting COVID-19: Yes vs. No	1.13 (0.73–1.74)	**1.80 (1.40–2.30)**
Fear of infecting the loved ones: Yes vs. No	0.73 (0.39–1.39)	**1.89 (1.22–2.89)**
Having a loved one infected with COVID- 19: Yes vs. No	1.26 (0.98–1.62)	**1.41 (1.22–1.63)**
Having a loved passed away due to COVID- 19: Yes vs. No	**1.78 (1.13–2.82)**	1.31 (0.98–1.74)
Feeling stigmatized or discriminated against as a health worker due to the pandemic: Agree vs. Disagree	**2.98 (2.36–3.77)**	**2.30 (2.01–2.64)**
Experienced violence due to being a health worker during the pandemic: Agree vs. Disagree	**4.72 (3.41–6.54)**	**2.85 (2.16–3.75)**
Having loved ones who support when needed: Agree vs. Disagree	**0.26 (0.20–0.35)**	**0.39 (0.31–0.49)**

Table 6 summarizes the results from regression analyses for workplace-level factors. Participants were more likely to show clinically significant depressive symptoms if they had spent up to 19 days [*OR* = 1.42: 95% CI (1.03–1.96)] or 20 or more days [*OR* = 1.52 95%CI (1.01–2.32)] in isolation due to COVID-19, or if they were assigned to a new team or functions [*OR* = 1.38: 95%CI (1.06–1.79)]. Furthermore, they were more likely to report clinically significant depressive symptoms if they were being close to patients who were suspected or confirmed cases of COVID-19 [*OR* = 1.39: 95% CI ([1.03–1.87)], if they had close contact at work with someone who later passed away due to COVID-19 [*OR* = 2.05: 95% CI (1.56–2.70)], or if they cared for patients with COVID-19 that then passed away [*OR* = 1.79: 95% CI (1.34–2.39).] In answering these three questions, even when respondents were not certain about the COVID-19 status of the patients or persons at work (in that period, it was not always possible to get COVID-19 tests in Italy, and many participants chose the option “I don't know”), they showed an increased risk of CSD. Having low levels of trust in the workplace capacity to manage COVID-19 increased the likelihood of reporting CSD [*OR* = 2.43: 95% CI (1.92–3.07)]. On the contrary, having a reliable network of supportive colleagues at work was a protective factor [*OR* = 0.31: 95% CI (0.24–0.39)].

**Table 6:** Association between health workers workplace-level factors and CSD (PHQ-9 ≥ 15) or GHQ + (GHQ-12 ≥ 4).

	**OR (95% CI)**	
	**CSD**	**GHQ+**
Hours worked per day on average (in the last week): • ≥ 10 vs. • ≤ 9	1.16 (0.87–1.54)	**1.41 (1.20–1.65)**
Days in isolation due to COVID: • 1–19 vs. 0 • ≥ 20 vs. 0	• **1.42 (1.03–1.96)** • 1.52 (1.00–2.32)	• **1.57 (1.30–1.90)** • **1.71 (1.32–2.21)**
Assignment to a new team and/or functions: Yes vs. No	**1.38 (1.06–1.79)**	**1.53 (1.31–1.78)**
Being close to patients who were suspected or confirmed cases of COVID-19: • Yes vs. No • I don't know vs. No	• **1.39 (1.03–1.87)** • **1.70 (1.27–2.27)**	• **1.65 (1.40–1.96)** • **1.53 (1.29–1.82)**
Close contact at work with someone who later passed away due to COVID-19: • Yes vs. No • I don't know vs. No	• **2.05 (1.56–2.70)** • **2.01 (1.41–2.86)**	• **1.90 (1.62–2.24)** • **1.98 (1.60–2.45)**
Having any of the patients with COVID-19 that respondent cared for passed away: • Yes vs. No • I don't know vs. No	• **1.79 (1.34–2.39)** • **1.69 (1.18–2.41)**	• **1.76 (1.50–2.07)** • **1.64 (1.33–2.01)**
Lack of masks from the beginning of the pandemic: Yes vs. No	1.17 (0.72–1.90)	1.13 (0.872–1.48)
Trust in the workplace capacity to manage COVID: Low vs. High	**2.43 (1.92–3.07)**	**1.99 (1.74–2.28)**
Having a reliable network of supportive colleagues at work: Agree vs. Disagree	**0.31 (0.24–0.39)**	**0.37 (0.32–0.43)**

### Association Between Healthcare Workers Individual- and Workplace-Level Factors and Psychological Distress

Table 5 summarizes the results from regression analyses on psychological distress for individual-level factors. Feeling stigmatized or discriminated against and experiencing violence due to being a HCWs during the pandemic increased the likelihood of reporting GHQ + [*OR* = 2.30 95% CI (2.01-2.64)] and *[OR* = 2.85: 95% CI (2.16-3.75)], respectively). Participants were more likely to report clinically significant psychological distress if they lived with persons older than 64 years [OR = 1.22: 95% CI (1.01–1.49)] or with disabilities [OR = 1.94: 95% CI (1.46–2.56)]. Furthermore, they were more likely to show clinically significant psychological distress if they reported fear of getting COVID- 19 [OR =1.80 95%CI (1.40–2.30)] or infecting the loved ones [OR= 1.89: 95% CI (1.22–2.89)], or if they had a loved one infected with COVID-19 [OR= 1.41: 95% CI (1.22–1.63)]. On the contrary, having loved ones who provided support when needed was a protective condition [OR =0.39 95% CI (0.31–0.49)].

Table 6 summarizes the results from regression analyses for workplace-level factors. Participants were more likely to show clinically significant psychological distress if they had spent up to 19 days [OR = 1.57 95% CI (1.30–1.90)] or 20 or more days in isolation due to COVID-19 [OR = 1.71 95% CI (1.32–2.21)], or if they were assigned to a new team or functions [OR = 1.53 95% CI (1.31–1.78)]. Furthermore, they were more likely to show clinically significant psychological distress if they were being close to patients who were suspected or confirmed cases of COVID-19 [OR = 1.65: 95% CI (1.40–1.96)], if they had close contact at work with someone who later passed away due to COVID-19 [OR = 1.90: 95% CI (1.62–2.240)], or if they cared for patients with COVID-19 that then passed away (OR = 1.76: 95% CI [1.50–2.07]). In answering these three questions, even when respondents were not certain about the COVID-19 status of the patients or persons at work, they showed an increased risk of psychological distress. Having low levels of trust in the workplace capacity to manage COVID-19 increased the likelihood of reporting GHQ + (OR = 1.99: 95% CI [1.74–2.28]). On the contrary, having a reliable network of supportive colleagues at work was a protective factor (OR = 0.37: 95% CI [0.32–0.43]).

## Discussion

The present study investigated the impact of the COVID-19 pandemic on the mental health of a large sample of Italian healthcare workers, analyzing the role of individual– and workplace–level factors in the reopening phase after the first, strict lockdown.

Among the individual-level factors examined, feeling stigmatized or discriminated against as a healthcare worker due to COVID-19 and having experienced violence due to being a healthcare worker during the pandemic were associated with both clinically significant depressive symptoms and psychological distress. It must be kept in mind that our study was conducted at the end of a period when HCWs were praised as “heroes”, and it was generally believed that the Italian community had gathered around them with great solidarity, while in other countries HCWs were being strongly discriminated and attacked (25, 26). Among several studies conducted during the COVID-19 first wave in Italy, only one in Lombardy assessed discrimination and violence experienced by HCWs, 25% reported episodes of discrimination against themselves, their colleagues, or their family members and even episodes of physical assault and vandalism. Our study demonstrates that during the reopening period after the first wave, discrimination and violence against HCWs were not isolated to specific “high-risk” areas but widespread throughout the national territory. Furthermore, they were strongly associated with clinically significant depressive symptoms and psychological distress among HCWs.

Numerous other individual-level factors were related to negative mental health outcomes. For instance, living with a person older than 64 years or with a disability was associated with clinically significant depressive symptoms and psychological distress. Older adults and persons with disabilities were found at higher risk of severe forms of COVID-19 (27, 28). The increased risk of infection of HCWs and the consequent risk of infecting their family members, coupled with the difficulty of adopting strategies such as physical distancing or isolation in situations where HCWs were also caregivers or supporters in family life, could explain the increased levels of psychological distress. Furthermore, being a caregiver or a supporter has been found to increase the risk of developing depression due to the constant demands caregivers need to face while providing care (29). This is even more the case during a pandemic when HCWs have to deal with additional demands not only at home but also at work. Fear of getting COVID-19, fear of infecting the loved ones, and having a loved one infected with COVID-19 were associated with clinically significant psychological distress but not depression. On the contrary, having a loved one pass away due to COVID-19 was associated with clinically significant depressive symptoms but not psychological distress. A relationship between the loss of a loved one and depression has been shown in several studies (30, 31). The COVID-19 pandemic has complicated such relationship by disrupting the usual experiences of grief. This situation led to increased levels of depression in people grieving loved ones passed due to COVID-19, including HCWs (32–34). Given that HCWs are also at higher risk of getting the infection and thus transmitting it, in many situations, they may have felt responsible for causing the death of their loved ones or experienced survivors guilt and this, in turn, may have led to depression (35). In contrast with the other variables examined, having loved ones able to support when needed was a powerful protective factor against depression and psychological distress among HCWs.

Almost all the workplace-level aspects we analyzed appear to be associated both with clinically significant depressive symptoms and psychological distress. Low trust in the workplace capacity to manage COVID-19 and having a reliable network of supportive colleagues at work were the factors with the strongest association. This is not a surprise considering that the financial cuts to the national health system left Italian health services with limited resources and personnel (8), and thus in a difficult position to face the pandemic effectively. This could also explain why the assignment to a new team and functions was associated with depressive symptoms in Italy but not, for instance, in a country such as Spain that, although close to Italy in terms of organization of services, did not experience the same lack of resources and personnel (36–38). When health services are understaffed, as in Italy, the changes of teams and functions are more frequent and stressful, and having a network of supportive colleagues at work becomes more critical to protect mental wellbeing (39). Interestingly, in contrast with previous research (40), our study found that working long hours was associated with psychological distress but not with depressive symptoms.

Another point worth mentioning is that our depressive symptoms and psychological distress estimates were lower than those of similar Italian and international studies conducted during the pandemic. We found that 7.55% of HCWs showed symptoms of clinically significant depressive symptoms and 37.89% of clinically significant psychological distress while other surveys carried out in Italy among health professionals reported estimates of depression in the whole sample ranging from 13.2% to 62% (41–44). A survey conducted within the framework of the COVID-19 HEROES initiative in Spain, a country close to Italy in terms of geography, culture, and organization of services, found that 27% of the HCWs interviewed screened positive at the PHQ-9 and 74% at the GHQ-12 (36). The lower estimates in our study can be explained by the fact that we collected data during the re-opening phase after lockdown, when the number of COVID-19 cases and thus the workload of HCWs were decreasing, while in Spain and the other studies cited data were collected during the first wave (36, 41, 42) or at the beginning of the second wave (43, 44). Furthermore, we decided to adopt a very high cut-off both for the PHQ-9 (scores ≥ 15) and the GHQ-12 (scores ≥ 4 using the bimodal scoring method), while for instance, the Spanish group adopted lower cut-offs (PHQ-9 > 9 and GHQ-12 > 2) (36). Even when applying the same cut-offs adopted in Spain, we still found lower, although closer, estimates (22.3% of the HCWs screening positive at the PHQ-9 and 45.74% at the GHQ-12). Scores of 15 or greater on the PHQ-9 have high specificity, though low sensitivity, for major depressive disorder (sensitivity: 0.56, specificity: 0.96, PPV: 51%) (21, 45). Similarly, scores ≥ 4 at the GHQ-12 using the bimodal scoring method indicate the likely presence of clinically significant psychological distress (24). Since we aimed to examine the association between individual and workplace-level factors and HCWs' mental wellbeing, we decided to adopt higher cut-offs to increase specificity and positive predictive value at the expense of sensitivity. Nevertheless, the prevalence of clinically significant psychological distress or depressive symptoms among the HCWs in our study is much higher than the prevalence of anxiety and depression found with less conservative cut-off scores in the general Italian population (46, 47) and in previous studies among Italian HCWs (48) before the pandemic.

Our study presents several strengths: the focus on the “reopening phase” after a strict lockdown, a phase of the pandemic overlooked in previous studies, the analysis of factors such as stigma and discrimination against HCWs previously thought peripheral in the Italian context, the inclusion of health professionals usually neglected in studies regarding the pandemic, such as non-clinical HCWs and HCWs employed outside the hospital system, the inclusion of HCWs from Italian areas differently affected by the pandemic (North, Center, and South), and the large sample size. However, our study is not without limitations. We used a non-probabilistic sampling approach, which may have hindered our findings' validity (49) and carries the risk of selection bias. The study's design was cross- sectional, and thus we cannot exclude reverse causation. In addition, we used self-report instruments and *ad-hoc* instruments that could introduce a bias in comparison with interview-based measures. However, we could not adopt a different methodology due to the COVID-19 restrictions at the time of data collection.

This study expands the literature on the psychological impact of the COVID-19 pandemic on healthcare workers. It shows that numerous individual and workplace-level factors were associated with clinically significant depressive symptoms and psychological distress among healthcare workers during the reopening phase after a strict lockdown. Our findings provide valuable information for policymakers, health service providers, and mental health professionals on the individual and workplace-level factors to target when developing and implementing interventions and preventive actions for future emergencies crises. In particular, the results of our study emphasize the need to address discrimination and violence against HCWs and improve healthcare work environments by eliminating or reducing risk conditions, enhancing social support by peers, and building trust in the healthcare institutions capacity to manage crisis situations in the future.

## Data Availability Statement

The datasets presented in this article are not readily available because of restrictions in the Ethical Committee approval. Data can be obtained by MFM and MGC upon reasonable request. Requests to access the datasets should be directed to mfmoro@gmail.com.

## Ethics Statement

The study was approved by the Ethics Committee of the Azienda Mista Ospedaliero-Universitaria of Cagliari, Italy. The research was conducted in compliance with the provisions that protect privacy in Europe (Articles 6 and 9 of EU Regulation No. 679). Informed consent was considered given when the health worker agreed to participate.

## Author Contributions

MM, ES, FM, and RA coordinated the international COVID-19 HEROES study. MM and MC coordinated the study in Italy and obtained funding. MM and MC conducted the analysis with input from ES and AR. MM drafted the paper and with input from MC, ES, FM, AR, RA, GC, RP, VD, AP, PK, FR, GL, EP, AP, FC, AU, and VR finalized the manuscript. All authors contributed to the article and approved the submitted version.

## Funding

The COVID-19 HEROES study in Italy was funded by the Italian Ministry of Education, Universities and Research (Bando FISR 2020IP_05308) and Fondazione di Sardegna (Bando 2020).

## Conflict of Interest

The authors declare that the research was conducted in the absence of any commercial or financial relationships that could be construed as a potential conflict of interest.

## Publisher's Note

All claims expressed in this article are solely those of the authors and do not necessarily represent those of their affiliated organizations, or those of the publisher, the editors and the reviewers. Any product that may be evaluated in this article, or claim that may be made by its manufacturer, is not guaranteed or endorsed by the publisher.
