# Supplementary material for: The Impact of the COVID-19 Pandemic on the Mental Health of Healthcare Workers in Italy: Analyzing the Role of Individual and Workplace-Level Factors in the Reopening Phase After Lockdown
Source: Front Psychiatry. 2022 Jun 2;13:867080. doi: 10.3389/fpsyt.2022.867080 (PMC9200968; doi:10.3389/fpsyt.2022.867080)
Supplement: Supplementary file 1 [file Data_Sheet_1.docx]

**Supplementary materials 1. Characteristics of the respondents: GHQ+ stratified by gender**

|  | **GHQ+ (%)** | **GHQ- (%)** | **Total (%)** | **OR (CI 95%)** |
| --- | --- | --- | --- | --- |
| **Man** | 386 | 872 | 1,258 (29.12) | --- |
| **Woman** | 1,250 | 1,808 | 3,058 (70.79) | **1.56 (1.36-1.89)** |
| **Other gender** | 0 | 4 | 4 (0.09) | ** |
| **Total** | 1,637 (37.89) | 2,683 (62.11) | 4,320 (100) |  |

**Not calculable

**Supplementary materials 2. Characteristics of the respondents: GHQ+ stratified by age**

|  | **GHQ+ (%)** | **GHQ- (%)** | **Total (%)** | **OR (CI 95%)** |
| --- | --- | --- | --- | --- |
| **≤30** | 330 | 471 | 801 (18.54) | --- |
| **31-43** | 501 | 770 | 1,271 (29.42) | 0.93 (0.78-1.11) |
| **44-56** | 507 | 852 | 1,359 (31.46) | 0.85 (0.71-1.01) |
| **57-69** | 290 | 573 | 863 (19.98) | **0.72 (0.59-0.88)** |
| **≥70** | 9 | 17 | 26 (0.60) | 0.76 (0.33-1.72) |
| **Total** | 1,637 (37.89) | 2,683 (62.11) | 4,320 (100) |  |

**Supplementary materials 3. Characteristics of the respondents: GHQ+ stratified by profession**

|  | **GHQ+ (%)** | **GHQ- (%)** | **Total** | **OR (CI 95%)** |
| --- | --- | --- | --- | --- |
| **Physician** | 615 | 920 | 1,535 (35.53) | --- |
| **Social Worker** | 0 | 5 | 5 (0.12) | ** |
| **Occupational Therapist / Educator /**  **Rehabilitation Technician** | 148 | 239 | 387 (8.96) | 0.93 (0.74-1.16) |
| **Physical therapist** | 126 | 230 | 356 (8.24) | 0.82 (0.64-1.04) |
| **Nurse** | 223 | 319 | 542 (12.55) | 1.05 (0.86-1.28) |
| **Speech Therapist** | 87 | 117 | 204 (4.72) | 1.11 (0.83-1.49) |
| **Dietician** | 9 | 18 | 27 (0.63) | 0.75 (0.33-1.68) |
| **Socio-sanitary operator** | 31 | 62 | 93 (2.15) | 0.75 (0.48-1.16) |
| **First responder (e.g., EMT)** | 3 | 10 | 13 (0.30) | 0.45 (0.12-1.64) |
| **Dentist** | 25 | 39 | 64 (1.48) | 0.96 (0.57-1.60) |
| **Midwife** | 6 | 9 | 15 (0.35) | 1.00 (0.35-2.82) |
| **Psychologist** | 24 | 56 | 80 (1.85) | 0.64 (0.39-1.04) |
| **Administrator / Secretary / Admissions / Patient information** | 32 | 83 | 115 (2.66) | **0.58 (0.38-0.88)** |
| **Clinical and non-clinical manager (director)** | 14 | 27 | 41 (0.95) | 0.78 (0.40-1.49) |
| **Laboratory Technician** | 60 | 121 | 181 (4.19) | 0.74 (0.53-1.03) |
| **Maintenance, food, and security staff** | 4 | 14 | 18 (0.42) | 0.43 (0.14-1.30) |
| **Cleaning staff** | 8 | 18 | 26 (0.60) | 0.66 (0.29-1.54) |
| **Radiology technician** | 46 | 80 | 126 (2.92) | 0.86 (0.59-1.25) |
| **Other staff** | 175 | 314 | 489 (11.32) | 0.83 (0.67-1.03) |
| **Total** | 1,636 (37.89) | 2,681 (62.11) | 4,317 (100) |  |

Missing data (n=3)

**Not calculable
